# CytoCluster: A Cytoscape Plugin for Cluster Analysis and Visualization of Biological Networks

**DOI:** 10.3390/ijms18091880

**Published:** 2017-08-31

**Authors:** Min Li, Dongyan Li, Yu Tang, Fangxiang Wu, Jianxin Wang

**Affiliations:** 1 School of Information Science and Engineering, Central South University, Changsha 410083, China; limin@csu.edu.cn (M.L.); tangyu@csu.edu.cn (Y.T.); faw341@mail.usask.ca (F.X.W.); 2 School of software, Central South University, Changsha 410083, China; dongyanli@csu.edu.cn; 3 Department of Mechanical Engineering and Division of Biomedical Engineering, University of Saskatchewan, Saskatoon, SK S7N 5A9, Canada

**Keywords:** biological networks, cluster analysis, cytoscape, visualization

## Abstract

Nowadays, cluster analysis of biological networks has become one of the most important approaches to identifying functional modules as well as predicting protein complexes and network biomarkers. Furthermore, the visualization of clustering results is crucial to display the structure of biological networks. Here we present CytoCluster, a cytoscape plugin integrating six clustering algorithms, HC-PIN (Hierarchical Clustering algorithm in Protein Interaction Networks), OH-PIN (identifying Overlapping and Hierarchical modules in Protein Interaction Networks), IPCA (Identifying Protein Complex Algorithm), ClusterONE (Clustering with Overlapping Neighborhood Expansion), DCU (Detecting Complexes based on Uncertain graph model), IPC-MCE (Identifying Protein Complexes based on Maximal Complex Extension), and BinGO (the Biological networks Gene Ontology) function. Users can select different clustering algorithms according to their requirements. The main function of these six clustering algorithms is to detect protein complexes or functional modules. In addition, BinGO is used to determine which Gene Ontology (GO) categories are statistically overrepresented in a set of genes or a subgraph of a biological network. CytoCluster can be easily expanded, so that more clustering algorithms and functions can be added to this plugin. Since it was created in July 2013, CytoCluster has been downloaded more than 9700 times in the Cytoscape App store and has already been applied to the analysis of different biological networks. CytoCluster is available from http://apps.cytoscape.org/apps/cytocluster.

## 1. Introduction

In recent years, people have paid more and more attention to recognizing life activities within a cell by protein interactions and protein complexes [1,2,3] in the field of systems biology. Proteins are one of the most important biological molecules in a cell. Within a cell, a protein cannot work alone, but rather works together with other proteins to perform cellular functions. Proteins are involved in a life process through protein complexes. Protein complexes can help us to understand certain biological processes and to predict the functions of proteins. Also, they can realize the cell signaling regulation functions by allosteric, competitive binding, interaction, and post-translational modification [4]. Protein-protein interaction (PPI) networks are powerful models that represent the pairwise protein interactions of organisms. Clustering PPI networks can be useful for isolating groups of interacting proteins that participate in the same biological processes or that, together, perform specific biological functions.

Up to now, many clustering algorithms, which are used to predict protein complexes from proteomics data, have been proposed and applied to biological networks. Out of these methods, the graph-based approaches are the most popular, which includes the partition-based clustering method, the density-based clustering method, the hierarchical-based clustering method and the spectral-based clustering method.

The partition-based clustering algorithms detect protein complexes by finding an optimal network partition, and making sure that the divided objects in the same cluster are as close as possible and the objects in different clusters are as far away as possible, such as HCS (Highly Connected Subgraph) [5], RNSC (Restricted Neighborhood Search Clustering) [6], MSCF (Minimal Seed Cover for Finding protein complexes) [7]. These partition-based clustering algorithms need to know the partition number, which is albeit generally unknown to us. What is more, partition-based methods cannot predict overlapping clusters.

The density-based clustering algorithms identify protein complexes by mining dense subgraphs from biological networks, such as MCL (Markov CLuster) [8], MCODE (Molecular COmplex DEtection) [9], CPM (Clique Percolation Method) [10], LCMA (Local Clique Merging Algorithm) [11], Dpclus (Density-periphery based clustering) [12], IPCA (Identifying Protein Complex Algorithm) [13], CMC (Clustering based on Maximal Cliques) [14], MCL-Caw (a refinement of MCL for detecting yeast complexes) [15], ClusterONE (Clustering with Overlapping Neighborhood Expansion) [16], and so on. These clustering algorithms have the advantage of recognizing dense subgraphs. However, it is difficult to predict the clusters which are non-dense subgraphs with these methods, such as the subgraph of “star” and “cycle.”

The basic idea of the hierarchical clustering method is measuring the possibility that any two proteins are located in the same cluster according to their similarity or the distance between them. Hierarchical clustering methods can be further divided into divisive methods and agglomerative methods. A divisive method is a top-down approach, whose main action regards the total PPI network as a cluster first, then divides the network according to a rule until all nodes belong to different clusters. An agglomerative method is a bottom-up approach, whose main action regards each protein in the PPI network as a cluster first, then merges any two clusters according to their similarity value until all nodes are assigned to clusters. For example, G-N (Girvan-Newman) [17], MoNet (Modular organization of protein interaction Networks) [18], FAG-EC (Fast AGglomerate algorithm for mining functional modules based on the Edge Clustering coefficients) [19], EAGLE (agglomerativE hierarchicAl clusterinG based on maximaL cliquE) [20], HC-PIN (Hierarchical Clustering algorithm in Protein Interaction Networks) [21] are all hierarchical clustering algorithms. Hierarchical clustering methods can be used for mining arbitrary shape clusters, and can render the hierarchical organization of the entire PPI network based on a tree structure. However, this type of method is very sensitive to noise data and cannot obtain overlapping clusters. Some researchers extend the hierarchical clustering method to detect overlapping clusters by initializing a triangle with three interacting proteins instead of a single protein, such as OH-PIN (identifying Overlapping and Hierarchical modules in Protein Interaction Networks) [22].

The spectral-based clustering algorithms predict protein complexes based on the spectrum theory, such as QCUT (Combines spectral graph partitioning and a local search to optimize the modularity Q) [23], ADMSC (Adjustable Diffusion Matrix-based Spectral Clustering) [24], and SSCC (Semi-Supervised Consensus Clustering) [25]. These spectral-based clustering methods can be a simple and fast approach to a certain extent. These clustering algorithms depend on the feature vector, which determines the final clustering results. In addition, many other kinds clustering algorithms can be found in survey papers [26,27].

With the developments of clustering methods, the visualization of clusters becomes more and more important. Several tools [28,29,30,31,32,33] have been developed to help researchers to better recognize positive protein complexes. Cytoscape [34] is a friendly and open bioinformatics platform, which shows an exceptional performance both in virtualizations and manipulation of biological networks. Cytoscape also has the advantage of formidable extensibility of integrating a vast amount of plugins with diverse functions over other platforms. There are 33 apps concerning clustering based on Cytoscape described in our supplement, many of which aim to find meaningful pathways, or visualize networks by semantic similarities, or construct dynamic networks. Among all of the apps, there are several apps, such as ClusterViz [35], clusterMake [36], and ClusterONE [16], which are used to detect and visualize protein complexes in PPI networks. They are all useful tools with different clustering methods, which have been used in different areas of life sciences in recent years. However, a great deal of newly developed clustering algorithms has lost favor with the Cytoscape platform and do not implement visualization. Also, several plugins with old versions cannot work on the new Cytoscape platform any more. In order to solve the above limitations, we developed a new plugin named CytoCluster, which integrates six new clustering algorithms in total. In our plugin, five new approaches named IPCA, OH-PIN, HC-PIN, DCU (Detecting Complexes based on Uncertain graph model) [37], IPC-MCE (Identifying Protein Complexes based on Maximal Complex Extension) [38] were added, which are not integrated in any existing apps, but are important methods used to predict protein complexes. Our CytoCluster plugin also contains the BinGO function, which is used to determine which Gene Ontology (GO) categories are statistically overrepresented in a set of genes or a subgraph of a biological network. So, our app becomes a versatile tool that offers such comprehensive clustering algorithms, in addition to the BinGO function for biological networks.

## 2. Architecture

In this paper, we adopt Cytoscape 3.x to develop our app. Cytoscape 3.x has notable advantages over Cytoscape 2.x, which can be described in the following two aspects. First, the platform of Cytoscape 3.x adopts the OSGI (Open Service Gateway Initiative) framework, which allows developers to dynamically install, load, update, unload, and uninstall the newly developed bundles in an easy way. Second, Cytoscape 3.x employs Maven, which can help developers manage many jar files. In Cytoscape 3.x, both core modules and apps are called OSGI bundles, and they can significantly reduce complexity in app development to some extent. Also, two methods can be used for developing apps in Cytoscape 3.x. The first way is to develop apps as bundles, which can both register a service in the OSGI framework and withdraw its service from the registry. The second way is to implement the apps with Simplified CyApp API (Application Programming Interface), just like in Cytoscape 2.x.

**Figure 1:**
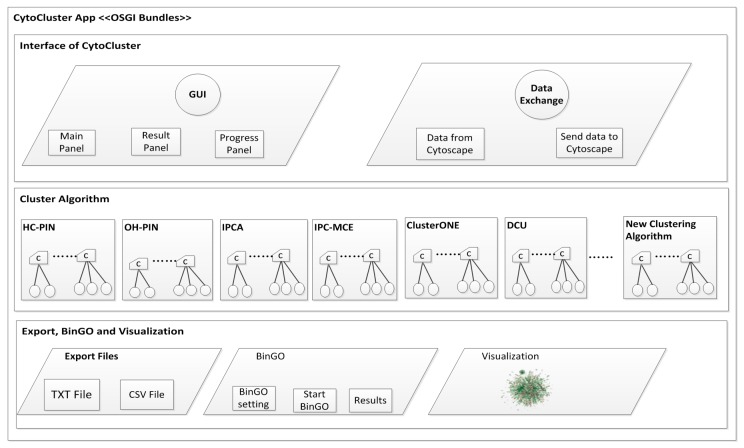
Architecture of CytoCluster.

The architecture of CytoCluster is shown in Figure 1, which includes three main bundles: the interface of CytoCluster bundles, the cluster algorithm bundles, and the visualization, BinGO, and export bundles. The interface of CytoCluster bundles is made up of a graphic user interface and a data exchange system, which allows the users to obtain different forms of bioinformatics networks including .txt and .csv files, and send the clustering results to Cytoscape. The six clustering algorithms bundles play an important role in our plugin CytoCluster, and we have defined the abstract Java class named clustering algorithms, making it is easy for us to integrate more clustering algorithms in CytoCluster. The BinGO bundles are the core functionality in analyzing the GO terms, which can be used to determine which GO categories are statistically overrepresented in a set of genes or a subgraph of a biological network. The visualization of BinGO and export bundles provide a way to intuitively visualize the clustering results in Cytoscape, determine which GO categories are statistically overrepresented, and export the clustering results to .txt or .cvs files.

## 3. Implementation

A user-friendly clustering software system to detect clusters is very important for biologists. By running the software, users can easily detect and analyze the protein complexes participating in the different life activities. Based on this basic idea, we developed our plugin CytoCluster by adopting the OSGI framework and the Cytoscape Maven archetypes. These frameworks and archetypes can create a maven-based project that builds an initial OSGI bundle-based Cytoscape app. The design is guided by the following three goals: first, to extend new clustering algorithms and add more functions; second, to dispatch the interface of CytoCluster and the algorithms; third, to respond quickly when the user operates the GUI (Graphical User Interface).

CyActivator class is an abstract class, which plays an important role in connecting Cytoscape with CytoCluster. All of the functions of CyActivator start to work as soon as you install the CytoCluster.jar for Cytoscape. The Analyze Action, as one of the service bundles, is the most important function in CytoCluster. Once the network is imported into Cytoscape, then our plugin CytoCluster is able to obtain these data from Cytoscape for further analysis. Two parts can be seen in the main panel. The top part mainly contains the two kinds of the clustering algorithms, overlap clustering algorithms and nonoverlap clustering algorithms. The bottom panel mainly provides six clustering algorithm panels, which are the IPCA panel, HC-PIN panel, OH-PIN panel, DCU panel, ClusterONE panel, and IPC-MCE panel. The user can choose different parameters according to their needs from these clustering algorithm panels. The result panel and the “export to .txt” function must be contained in CytoCluster, which provides an easy way to further analyze the results produced by different clustering algorithms. In addition, the progress panel is included in our app, which is used to visualize the progression of the running clustering algorithms.

Finally, we constructed this CytoCluster app containing four parts: Open, Close, About, and BinGO. Each part has its own function. Six clustering algorithms are included in the Open part. When users want to terminate this app, they should select the Close part. Here, BinGO plays an important role in determining which GO categories are statistically overrepresented in biological networks. Lastly, if you want to learn more information about the app, you cannot miss the About part.

### 3.1. Calculation and Basic Analysis

When users open the CytoCluster plugin, six clustering algorithms are provided, which are HC-PIN, OH-PIN, IPCA, IPC-MCE, ClusterONE, and DCU. In the following, these six clustering algorithms are briefly described.

#### 3.1.1. HC-PIN (Hierarchical Clustering Algorithm in Protein Interaction Networks)

The HC-PIN algorithm [21] is a fast, hierarchical clustering algorithm, which can be used in a weighted graph or an unweighted graph. The main processes can be described as follows. First, all vertices in the PPI network are regarded as singleton clusters. Then, HC-PIN [21] calculates the clustering value of each edge and queues all of the edges into a queue Sq in non-increasing order according to their clustering values. The higher clustering value the edge has, the more likely its two vertices will be in the same module. In the process of adding edges in the queue Sq to cluster, λ-modules are formed. Finally, λ-modules can be outputted when the number of its proteins is no less than a threshold *s*.

#### 3.1.2. OH-PIN (Identifying Overlapping and Hierarchical Modules in Protein Interaction Networks)

The OH-PIN algorithm [22] is an improved hierarchical clustering method, which can identify overlapping clusters. The basic idea of OH-PIN can be summarized as follows. At the beginning, the cluster set C_set is empty. For each edge in the protein interaction network, its B_Cluster is generated and the B_Cluster is added to the C_set, if B_Cluster is not already included in the C_set, until every B_Cluster is included. Then, OH-PIN [22] merges all highly overlapping cluster pairs in the C_set in terms of the threshold overlapping value. After the above step, OH-PIN assembles all of the clusters in the C_set into λ-modules by gradually merging the cluster pair with the maximum clustering coefficient.

#### 3.1.3. IPCA (Identifying Protein Complex Algorithm)

The IPCA algorithm [13] is a density-based clustering algorithm, which can identify dense subgraphs in protein interaction networks. IPCA has four major sub-algorithms: weighting vertex, selecting weed, extending cluster, and extend-judgment. First, IPCA [13] calculates the weight of each edge by counting the common neighbors of its connected two nodes and computes the weight of each node by summing up the weights of its incident edges. The higher weight one node has, the more likely the node is regarded as the seed. At the beginning, a seed is initialed as a cluster. IPCA extends a cluster by adding vertices recursively from its neighbors in terms of the nodes’ priority. Whether a node can be added to a cluster is determined by two conditions: its interaction probability and the shortest path between it and the nodes in the cluster.

#### 3.1.4. IPC-MCE (Identifying Protein Complexes based on Maximal Complex Extension)

The IPC-MCE algorithm [38] is a maximal clique-based clustering algorithm. The basic idea of IPC-MCE can be described as follows. First, IPC-MCE removes all the nodes which have only one neighbor. Then IPC-MCE enumerates all the maximal cliques in the remained PPI network and puts them into the set MCS (Maximal Clique Sets). For each neighborhood vertex *v* of the maximal clique *K* in set MCS, if ${\mathrm{IP}}_{\mathrm{vk}}$ is no less than the threshold *t*, the vertex *v* can be added to the maximal clique *K*. The definition of IPv_k_ is as follows:

(1) $${\mathrm{IP}}_{\mathrm{vk}}=\frac{\left|E_{\mathrm{vk}}\right|}{\left|V_{k}\right|}$$

$E_{\mathrm{vK}}$ is the number of the edges between the vertex *v* and *K*, and |*V_k_*| is the number of nodes in K. Finally, IPC-MCE [38] filters the repeated maximal clique according to a pre-defined overlapping value.

#### 3.1.5. ClusterONE (Clustering with Overlapping Neighborhood Expansion)

The ClusterONE algorithm [16] mainly contains three steps. First, groups are grown by adding or removing vertices with high cohesiveness from selected seed proteins. At the beginning, the protein with the highest degree is regarded as the first seed and grows a cohesive group from it using a greedy procedure. ClusterONE repeats this grown process to form overlapping complexes until there are no proteins remaining in the PPI network. Then ClusterONE merges the highly overlapping pairs of locally optimal cohesive groups according to a pre-defined overlapping score. Finally, ClusterONE outputs protein complexes that contain no less than three proteins or whose density is larger than a given threshold ∂ (its default value is 0.8).

#### 3.1.6. DCU (Detecting Complexes Based on Uncertain Graph Model)

The DCU algorithm [37] is a clustering algorithm, which detects protein complexes based on an uncertain graph model. First, DCU [37] starts from a seed vertex and adds other vertices by using a greedy procedure to form a candidate core with high cohesion and low coupling. Then, DCU uses a core-attachment strategy to add attachments to core sets to form complexes. Specifically, for each protein of a candidate set, if its internal absolute degree is less than its external absolute degree, which consists of neighbors of protein vertices in the candidate set, the protein must be removed from the candidate set. Finally, DCU needs to solve the problem of the repeated protein complexes by controlling their overlapping value. Users can select any kind of clustering algorithms they want in the main panel and input the parameters of the algorithm, which decide the creation of a specific clustering algorithm object in memory. Our CytoCluster plugin also provides the visualization of clustering results after running each of these six clustering algorithms, which can be seen in the result panel in the form of a thumbnail list. They can be sorted by the score, the size, or the modularity. In the result panel, the “Export” button and “Discard Result” button are included. The “Export” button is used for exporting results to a .txt file, including the name of algorithm, the parameters, and the clusters, while the “Discard Result” button is used for closing the result panel. Users can close the visualization of clustering results after running these six clustering algorithms with default parameters. In addition, users can see the visualization of cluster results after running a clustering algorithm. Therefore, CytoCluster is a convenient and fast app to obtain smaller networks from a large network.

### 3.2. BinGO

Here, we integrate the BinGO function to be the part of the CytoCluster. All this is done for the convenience of the users. When they install a cytocluster jar, users can not only choose different clustering algorithms, but also use BinGO. Once the BinGO part is opened, a panel will appear in the center of the computer monitor. Users can make a choice from this setting panel according to their need. The main function of BinGO is to determine the overrepresentation of Gene Ontology (GO) categories in a subgraph of a biological network or a set of genes. Once given a set of genes or a subgraph of a network on the GO hierarchy, BinGO can map the predominant functional themes and output this map in the form of a Cytoscape graph. The BinGO function has the same features as the BiNGO [39] plugin. These features contain graphs or genes list inputs; make and use custom annotations, ontologies, and reference sets; save the extensive results in a tab-delimited text file format; and so on. Selecting the “Start BiNGO” button is required after users have chosen their basic parameters. Then, the visualization of GO can be seen from a chosen network. The result can also be saved in a .bgo, which can be used for further studies.

In the BinGO part, two modes are included for selecting the set of genes to be functionally recommended. One is the default mode, and the other is the flexible mode. In the default mode, nodes can be chosen from a Cytoscape network, either manually or by other plugins. In the flexible mode, nodes can be selected from other sources, for example a set of nodes that are obtained from an experiment and pasted in a text input box. Here, the relevant GO annotations can be retrieved and propagated upwards through the GO hierarchy; namely, any genes related to a certain GO category can be predicted explicitly and included in all parental categories. Two statistical tests are also concerned so as to assess the enrichment of a GO term better. The most important characteristic of the BinGO part is its interactive use for molecular interaction networks, such as protein interaction networks. Furthermore, it is very flexible for BinGO to use ontologies and annotations. Both the traditional GO ontologies and the GOSlim ontologies are supported by BinGO. Then, the Cytoscape graph produced by BinGO can be seen, altered, and saved in a variety of ways.

## 4. Cases Studies

CytoCluster integrates different types of clustering algorithms including density-based clustering algorithms, hierarchical clustering algorithms, and maximal clique-based methods. Many researchers have downloaded and used the plugin since CytoCluster was released. So far, CytoCluster has been downloaded more than 9700 times since it was released in July 2013. Several important scientific articles indicated that CytoCluster can help scholars with their studies on the mechanisms of biological networks. There are several generic stages of how to run the clustering algorithms in our CytoCluster plugin, which include installing the CytoCluster app, loading the network, setting the data scope and parameters of clustering algorithms, running the cluster algorithm, and receiving or exporting the information of clustering results. The “CytoCluster” menu appears in the “App” menu, after installing the CytoCluster app. In this paper, we present a case to illustrate the use of our plugin. In addition, more cases on these six clustering algorithms can be seen in Table 1.

**Figure 2:**
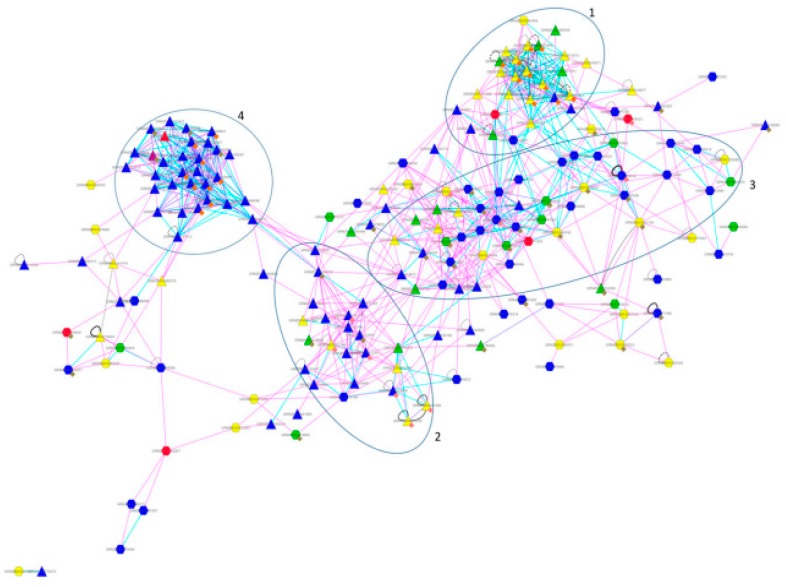
Four subnetworks achieved in the first case [58].

The case of CytoCluster was applied in botany [58]. This paper was published in Plant Physiology by Baute et al. The co-expression network was generated by Cytoscape 3.2.0 [59] according to the nodes and edges [60,61] at first. Then, the newly co-expression network was loaded, which incorporated 185 genes and 943 edges. Third, the main panel of the CytoCluster was opened and the HC-PIN clustering algorithm was chosen with standard settings and a complex size threshold of 10. In this case, 185 genes and 943 edges were included after dealing with the whole network. The identified subnetworks were further filtered, so as to only include the co-expression networks based on PCCs (Pearson Correlation Coefficients) of 0.7 and higher, as well as protein-protein interactions between query genes based on both experimental and predicted data from CORNET, when the users clicked on the “Analysis” button. Then, four subnetworks were formed after using our plugin for analysis, which can be seen from Figure 2. Each circle in Figure 2 shows a subnetwork. What is more, the generated co-expression network achieved by the HC-PIN algorithm can be seen in the result panel or exported to a .txt, so users can output the results from the different algorithms for further analysis. The table panel can list proprieties of clustering results when users select the corresponding clustering. The progress panel is used to visualize the progression of a specific cluster algorithm.

## 5. Conclusions

Our CytoCluster plugin is a platform-independent app for Cytoscape, which is also a functional diversity tool to offer different types of clustering algorithms, including IPCA, DCU, HC-PIN, OH-PIN, IPC-MCE, and ClusterONE. OH-PIN and HC-PIN are both hierarchical-based clustering algorithms, HC-PIN generates non-overlapping clusters, and on the contrary, OH-PIN produces overlapping clusters. IPCA, DCU, IPC-MCE, and ClusterONE are all density-based clustering algorithms, but the clusters generated by them also have some differences. Moreover, the same method will produce different results by changing the values of parameters. Users can both choose different clustering algorithms and analyze which GO categories are statistically overrepresented in a set of genes or a subgraph of a biological network. Our CytoCluster plugin is not only convenient for researchers to use, but also renders the investigated biological process easy to understand. Because our app has the advantage of expandability, more clustering algorithms such as those reported in References [62,63,64,65] as well as modules can be added to CytoCluster. Owing to such features, we firmly believe our app will be of great help in biology research.

## Figures and Tables

**Table 1:** More applications of CytoCluster and the six clustering algorithms integrated in it.

Algorithms	Application	Network	Description	Reference
IPCA	Exploring tomato gene functions	The tomato co-expression network was chosen and 465 complexes were found	IPCA was used to identity a densely connected network	[40]
	Unravelling gene function	The tomato co-expression network was chosen and 465 complexes were found	IPCA was choosen to identify thick connected nodes	[41]
	Predicting colon adenocarcinoma	The networks from IntAct and reactome were merged	IPCA was used to identify highly connected subnetworks	[42]
	The correlation between cold and heat patterns	The network from RA 18 was diagnosed with defciency pattern and 15 others were diagnosed with nondefciency pattern	IPCA was used to analyze the characteristics of networks	[43]
	Evidence-based complementary and alternative medicine	PPI network from genes was chosen so that the ratio of cold patterns to heat patterns in patients with RA was more or less than 1:1.4	IPCA was used to detect highly connected subnetworks	[44]
	Cold and heat patterns of rheumatoid arthritis	PPI network from these genes was chose that the ratio of cold patterns to heat patterns in patients with RA was more or less than 1:2	Highly connected regions associated with typical TCM cold patterns and heat patterns were identified	[45]
	Cold and heat pattern of rheumatoid arthritis	Network for differentially expressed genes between RA patients with TCM cold and heat patterns	IPCA was used to infer significant complexes or pathways in the PPI network	[46]
	Functional networks	Network contained some gene expressions or regulated proteins	Then eight highly connected regions were found by IPCA to infer complexes or pathways	[47]
	The molecular mechanism of interventions	PPI networks of biomedical combination was chosen and 11 complexes were found	IPCA was used to analyze the characteristics of the network	[48]
	The synergistic sechanisms	Network associated with Salvia miltiorrhiza and Panax notoginseng	Significant complexes or pathways were inferred	[49]
HC-PIN	Constraints on community	Associations between bacteria OTUs and four subnetworks were found	Subnetworks of OTUs were detected	[50]
	Strategies between two reef building cold-water coral species	Association network of the cold-water scleractinian corals bacterial communities	HC-PIN was used to identify OTUs	[51]
	Biomarkers	The network was extracted from the TCGA database	miRNA-gene clusters were identified	[52]
	Finding the candidate biomarkers for POAG disease	Network was extracted from previous studies with 474 proteins and nine subnetworks were found	HC-PIN was choosen to perform the clustering with a complex size threshold of 3	[53]
OH-PIN	Bacterial associations	Bulk soil DNA was extracted	The subnetworks were partitioned into modulars	[54]
ClusterONE	A census of human soluble protein complexes	Network was extracted from human HeLa S3 and HEK293 cells grown	ClusterONE was used to detect protein complexes	[55]
	An arabidopsis	A network with 8900 nodes and 6382 edges was chosen and 701 clusters were found	ClusterONE was used to obtain subnetworks	[56]
	Fndinge disease-drug modules	Disease-gene and drug-target associations were found from drug-target data	Overlapping subnetworks were identified	[57]

PPI: Protein-protein interaction; IPCA: Identifying Protein Complex Algorithm; TCM:Traditional Chinese Medicine; RA:Rheumatoid Arthritis; POAG: Primary Open Angle Glaucoma; OTU: Opearating Taxonomic Unit; TCGA:The Cancer Genome Atlas; OH-PIN: Identifying Overlapping and Hierarchical Modules in Protein Interaction Networks.
